# Worse Clinical and Survival Outcomes in Breast Cancer Patients Living in Puerto Rico Compared to Hispanics, Non-Hispanic Blacks, and Non-Hispanic Whites from Florida

**DOI:** 10.1007/s40615-024-02232-5

**Published:** 2024-11-14

**Authors:** Abigail E. Lantz, Ryan Gebert, Jiannong Li, Jose A. Oliveras, Edna R. Gordián, Jaileene Perez-Morales, Steven Eschrich, Dung-Tsa Chen, Marilin Rosa, Julie Dutil, Harold I. Saavedra, Teresita Muñoz-Antonia, Idhaliz Flores, William D. Cress

**Affiliations:** 1 Puerto Rico Biobank, H. Lee Moffitt Cancer Center & Research Institute, 12902 Magnolia Drive, Tampa, FL USA; 2 Department of Molecular Oncology, H. Lee Moffitt Cancer Center & Research Institute, Tampa, FL USA; 3 Department of Biostatistics and Bioinformatics, H. Lee Moffitt Cancer Center & Research Institute, Tampa, FL USA; 4 Department of Basic Sciences, Ponce Research Institute, Ponce Health Sciences University, Ponce, Puerto Rico; 5 Department of Cancer Epidemiology, H. Lee Moffitt Cancer Center & Research Institute, Tampa, FL USA; 6 Department of Anatomic Pathology, H. Lee Moffitt Cancer Center & Research Institute, Tampa, FL USA; 7 Ponce Health Sciences University, Ponce, Puerto Rico

**Keywords:** Cancer health disparities, Risk factors, Cancer outcome, Family cancer history, Hormone receptors, Cancer survival

## Abstract

**Background:**

Herein, we report the characterization of four cohorts of breast cancer patients including (1) non-Hispanic Whites in Florida, (2) non-Hispanic Blacks in Florida, (3) Hispanics in Florida, and (4) Hispanics in Puerto Rico.

**Methods:**

Data from female breast cancer patients were collected from cancer registry (*n* = 9361) and self-reported patient questionnaires (*n* = 4324). Several statistical tests were applied to identify significant group differences.

**Results:**

Breast cancer patients from Puerto Rico were least frequently employed and had the lowest rates of college education among the groups. They also reported more live births and less breastfeeding. Both Hispanic groups reported a higher fraction experiencing menstruation at age 11 or younger (Floridian Hispanics [38%] and Puerto Ricans [36%]) compared to non-Hispanic Whites (20%) and non-Hispanic Blacks (22%). Non-Hispanic Black and Puerto Rican women were significantly older at breast cancer diagnosis than their non-Hispanic White and Floridian Hispanic counterparts. The Puerto Rican and non-Hispanic Black groups more frequently had pathology stage T2 or higher primary breast tumors at diagnosis (non-Hispanic Whites [29%], non-Hispanic Blacks [39%], Floridian Hispanics [33%], Puerto Ricans [46%]). The Puerto Rican (73%, 95% CI [66, 82]) and non-Hispanic Black (79%, 95% CI [75, 84]) groups demonstrate reduced 5-year survival compared to non-Hispanic Whites (89%, 95% CI [86, 92]) and Floridian Hispanics (89%, 95% CI [86, 90]).

**Conclusions:**

These findings demonstrate that Puerto Rican breast cancer patients suffer significant breast cancer health disparities relative to non-Hispanic Whites and Hispanics from Florida similar to the disparities observed for non-Hispanic Blacks. Future work must seek to better understand and address these disparities.

**Supplementary Information:**

The online version contains supplementary material available at 10.1007/s40615-024-02232-5.

## Introduction

The Puerto Rico Biobank (PRBB) was established as a component of the NCI-funded *Partnership to Address Cancer Health Equity* (PACHE) between Ponce Health Sciences University (PHSU) in Ponce, Puerto Rico, and the Moffitt Cancer Center (MCC) in Tampa, Florida, to address the significant under-representation of Hispanic/Latinos (H/L), especially from the Caribbean region, in public cancer databases [1]. Despite the fact that we [2], and others [3–5] have reported significant differences in the frequency of mutations driving cancer among H/Ls related to their genetic ancestry, only 3% of data in the Cancer Genome Atlas, a large genomic study that is intended to be representative of the general population, is derived from H/L patients [6]. This percentage is substantially lower than the proportion of H/Ls within the United States (US) population which was estimated at 18.7% in the 2020 US Census (www.census.gov). Since 2008, the PRBB has contributed to filling this gap by serving as a biospecimen and data resource for investigators conducting cancer-related health disparities research. The PRBB consenters identify potential participants during the admission process for oncological surgeries and accrue biospecimens (tissues, biopsies, blood) and data (clinical, pathological, demographic) in collaboration with physicians (surgeons, oncologists, pathologists) in the Southern Puerto Rico region. Biospecimens (tissues and blood) are annotated, processed, and stored for future use to support a wide variety of biomedical research projects. Since 2008, the PRBB has consented over 4000 subjects and has collected over 9000 biospecimens. Tissue and data, including self-reported data through a comprehensive questionnaire and cancer registry data, have been released to support ~ 50 collaborative research projects aiming to characterize the genetic and molecular profiles of tumors from Puerto Rican patients. Breast cancer patients are the most frequently consented patients in the PRBB, and breast cancer is also the most commonly diagnosed cancer type in Puerto Rican women [7].

Breast cancer is the leading cause of cancer death among non-Hispanic Black (NHB) [8] and Hispanic/Latino women in the US [9]. Some studies indicate that relative to non-Hispanic White (NHW) women with breast cancer, H/L and NHB women have significantly poorer survival outcomes [10–15], while others find that H/L have better prognostic outcomes than NHW women with breast cancer [16, 17]. Several lifestyle factors, including obesity, smoking, and alcohol consumption are associated with a higher risk of developing breast cancer while physical activity is associated with lower risk [18]. Numerous demographic and clinical factors also likely contribute to these mortality and survival disparities, including socioeconomic status, healthcare access, the proportion of hormone receptor-negative (estrogen and progesterone receptor-negative, or ER-PR-) breast cancers, and tumor stage. For instance, there are differences in tumor characteristics among racial and ethnic groups, with a higher likelihood of hormone receptor-negative breast cancers in H/L and NHB women relative to NHW women, as well as a tendency for breast cancers to be detected at later (non-localized) stages in H/L and NHB women compared with NHW women [8, 12, 13, 15, 19–29]. Among H/Ls, women from Puerto Rico, Cuba, and the Dominican Republic represent a unique subgroup with higher breast cancer incidences [30, 31] and poorer survival outcomes than other US H/L [32, 33]. In a study that focused on H/L subgroups in the US [11], Puerto Rican women were found to have the highest mortality, the highest risk of presenting with late-stage disease, and the most likely to present with ER-/PR- disease than the other H/L subgroups studied.

The goal of this study is to describe a cohort of Puerto Rican women with breast cancer who have consented to donate tissues and data for research through the PRBB from 2009 to 2019. This cohort consists primarily of patients who will undergo oncologic surgery or who are receiving oncologic care from private practitioners in hospitals or clinics located in the Ponce Health Region (PHR), consisting of 15 towns around the city of Ponce in Southern Puerto Rico [7]. Per the State Data Center of Puerto Rico (https://censo.estadisticas.pr/), the PHR has a total population of 474,243 with 98.8–99.8% of its inhabitants self-identified as H/Ls. The PHR communities experience unique challenges, from patient-level limitations related to socioeconomic status and geographical context (rural, remote, mountainous areas) to structural problems inherent to the healthcare system (fragmented services, lack of support resources). In addition, in recent years, this region has been strongly impacted by natural disasters, including Hurricane Maria in 2017, the January 2020 earthquakes in southern PR, and Hurricane Fiona in 2022. Notably, the PHR has the highest rate of cancer mortality (104.2/100,000) of all PR regions according to the Puerto Rico Central Cancer Registry (PRCCR) (http://rcpr.org/) [7].

Our approach was to compare clinical and demographic data associated with female breast cancer patients collected from the PRBB (HPR) databases to their counterparts from the Moffitt Cancer Center (MCC) located in Tampa, FL, US. Through the analysis of these multi-sourced data, we hope to contribute to a better understanding of female breast cancer patients in the PHR, differences and/or similarities with other races and ethnicity groups, as well as demonstrate the need for acknowledging the heterogeneity of clinical presentation of cancer in different H/L populations and the structural limitations in access to high-quality care resulting in cancer-related health disparities.

## Methods

### Data Availability

Individual patient data supporting all analyses in the paper are available through the original sources described below.

### Patient Research

Patients provided written consent to participate in this study under one of four IRB-approved protocols: (PRI-IRB 080121-IF, MCC 20004/IRB 33379, MCC 21040/IRB 48234, or MCC 14690/IRB 104189). Self-reported data from questionnaires were collected from the PRBB and MCC databases. The abstracted PRBB questionnaires represent data collected from 2009 to 2019; data from MCC cases were collected from 2012 to 2020. Data captured by the PRBB and MCC questionnaires includes socio-demographic variables, risk factors and exposures, family history of cancer, and clinical history (cancer and other comorbidities, treatments, age at diagnosis, and specific symptomatology). Attrition was not documented, as we focused on questionnaire data collected at a single time point for each patient. Randomization and blinding were not performed, as this study was primarily descriptive. After excluding duplicate patient records, males, non-breast tumor sites, survey versions that did not contain our questions of interest, and incomplete questionnaires, the complete analytical data set of self-reported questionnaires included 4324 patients with breast cancer (Supplemental Fig. 1). The dataset was further divided into four race and ethnicity groups: NHW, NHB, Hispanics in Florida (HF), and HPR.

Independent of questionnaire completion, breast cancer hormone receptor subtype data for the Florida groups were abstracted from MCC clinical data sources. After excluding patients with incomplete or inconclusive estrogen receptor, progesterone receptor, and HER2 test results, as well as excluding duplicate patient reports, 9361 unique patient observations remained (Supplemental Fig. 2). The corresponding hormone receptor data for the Puerto Rican patients were abstracted from the PRCCR. Hormone receptor (HR) status was considered positive if ER and/or PR were positive and was considered negative if both ER and PR were negative. Age at diagnosis data was provided for MCC patients by the Florida Cancer Data System via the Collaborative Data Services Core at MCC, and the date at diagnosis was provided for HPR patients by the PRCCR. Age at diagnosis was calculated for HPR patients using patient-provided dates of birth from the PRBB questionnaires.

### Statistical Analysis

Variables for analysis were determined based on their relevance to breast cancer and being present in both study questionnaires (PRBB and MCC). Frequencies of various demographic, female reproductive health, family cancer history, hormone receptor, and pathological tumor stage variables were compared and analyzed using chi-squared tests and the Benjamini–Hochberg procedure to determine significant differences between the race and ethnicity groups. BMI and age at diagnosis data were analyzed using descriptive statistics, one-way ANOVA, and Tukey test to characterize each race and ethnicity group and any significant differences. Survival was analyzed using the log-rank test. All statistical analyses were performed in R version 4.3.2 [34].

## Results

### Demographics

**Fig. 1:**
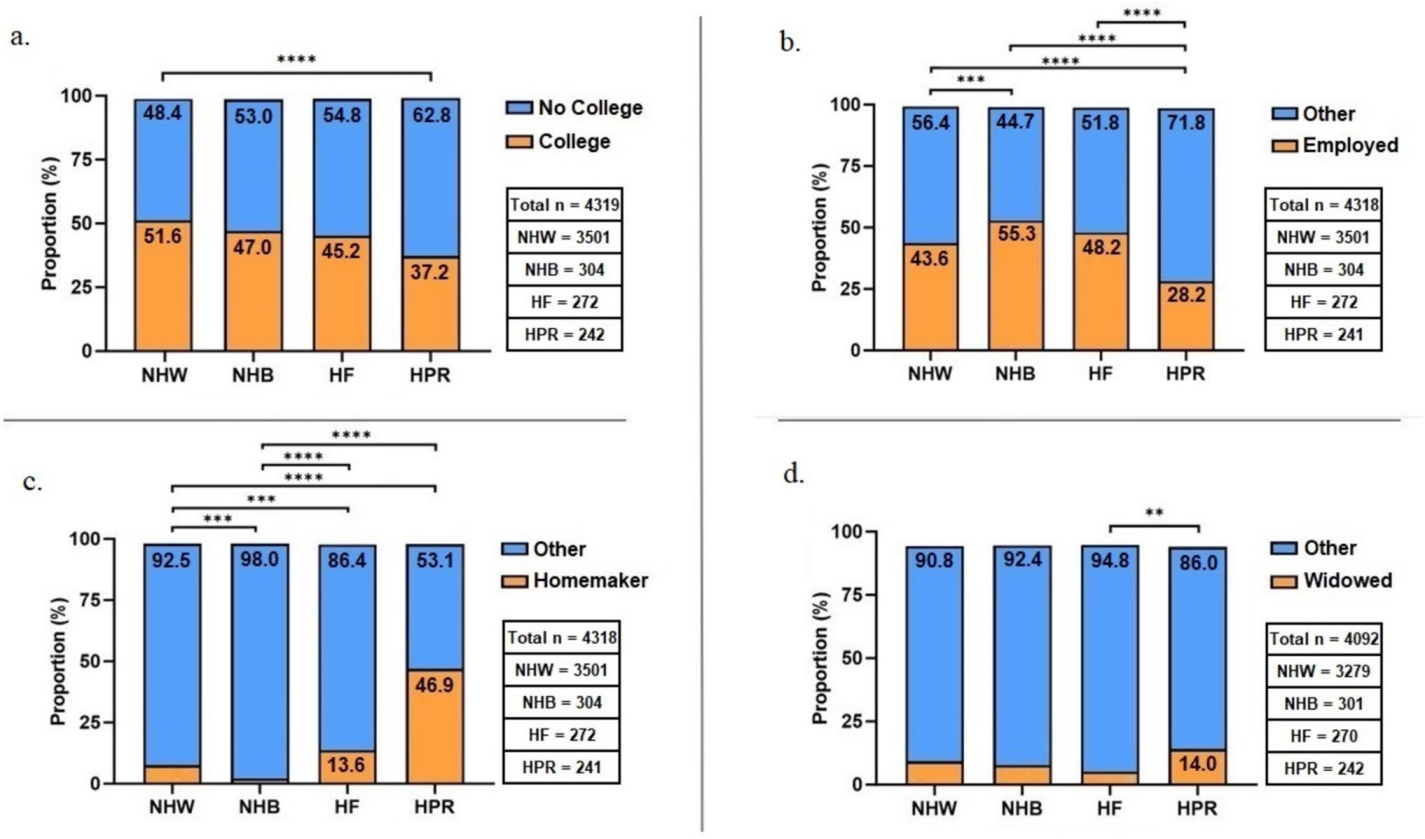
Patient demographic characteristics by cohort: college education (**a**), employment status (**b**), homemaking status (**c**), widowhood (**d**). Significant group differences were determined via chi-squared test and are indicated as **p < 0.01, ***p < 0.001, ****p < 0.0001

Figure 1 compares the four cohorts concerning education, employment, and marital status. In group-to-group comparisons, significantly (*p* < 0.0001) fewer HPR (37.2%) women were college-educated compared to the NHW group (51.6%). Similarly, HPR patients report being less frequently employed (28.2%) than women in any of the other groups (NHW [43.6%], HF [48.2%], and NHB [55.3%]). Likewise, the HPR women were more frequently homemakers (46.9%; *p* < 0.0001) than NHW women (7.5%) and NHB women (2.0%). Finally, women in the HPR cohort are widowed at a significantly higher frequency (14.0%; *p* < 0.01) than women in the HF group (5.25%). A full description of demographic data can be found in Supplemental Table 1. Differences in patient numbers (*N*) in each comparison are the result of missing values for some patients in this and subsequent analyses.

**Table 1:** Five-year overall survival rates by cohort

Five-year survival	NHW *N* = 17,678	NHB *N* = 1709	HF *N* = 1950	HPR *N* = 230
Min. confidence interval (%)	86	75	86	66
Median survival interval (%)	89	79	89	73
Max. confidence interval (%)	92	84	90	82

### OB-GYN Profiles

**Fig. 2:**
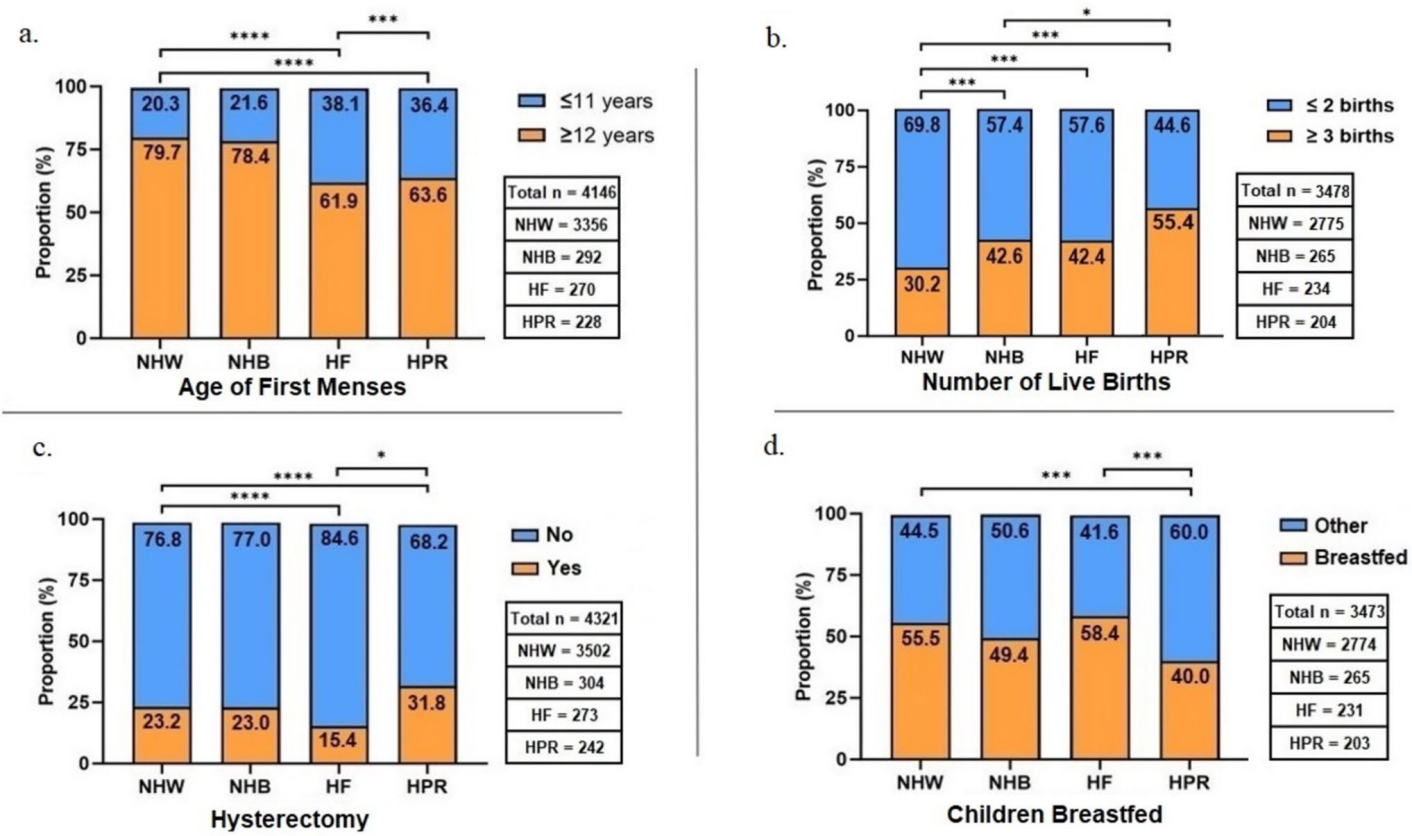
Patient OB-GYN variables by cohort: age at menarche (**a**), live births (**b**), hysterectomy status (**c**), breastfeeding history (**d**). Significant group differences were determined via chi-squared test and are indicated as *p < 0.05, ***p < 0.001, ****p < 0.0001

Figure 2 summarizes comparisons of patient OB-GYN variables by cohort. Each Hispanic cohort was found to have significantly higher rates of menarche at 11 years of age or younger than each non-Hispanic cohort. A percentage of 38.1% of HF women reported experiencing menarche before or at 11 years of age, which was found in a pairwise comparison to be significantly higher than the 21.6% of NHB women (*p* < 0.0001) and 21.2% of NHW women (*p* < 0.0001) reporting the same. HPR women reported early menarche at 36.4%, which was also found to be significantly higher than NHB (*p* < 0.001) and NHW (*p* < 0.0001) women. Significant differences were found in the proportion of women who reported having three or more live births: 55.4% of HPR patients compared to 42.6% of NHB patients (*p* < 0.05) and 30.2% of NHW patients (*p* < 0.001). Among the three Florida cohorts, NHW patients had significantly fewer live births than NHB patients (*p* < 0.001) and HF (*p* < 0.001) patients. HPR women reported the highest rate of hysterectomy (31.8%) among the cohorts, compared to 23.2% of NHW women (*p* < 0.0001) and 15.4% of HF women (*p* < 0.05). The HF group was found to have significantly lower rates of hysterectomy on pairwise comparison with the NHW group (*p* < 0.0001). Considering patients who had one or more live births, HPR women least frequently reported having breastfed at 40.0%, compared to their parous NHW (55.5%, *p* < 0.001) and HF (58.4%, *p* < 0.001) counterparts. A more complete description of OB-GYN profiles can be found in Supplemental Table 2.

### Family Cancer History

**Fig. 3:**
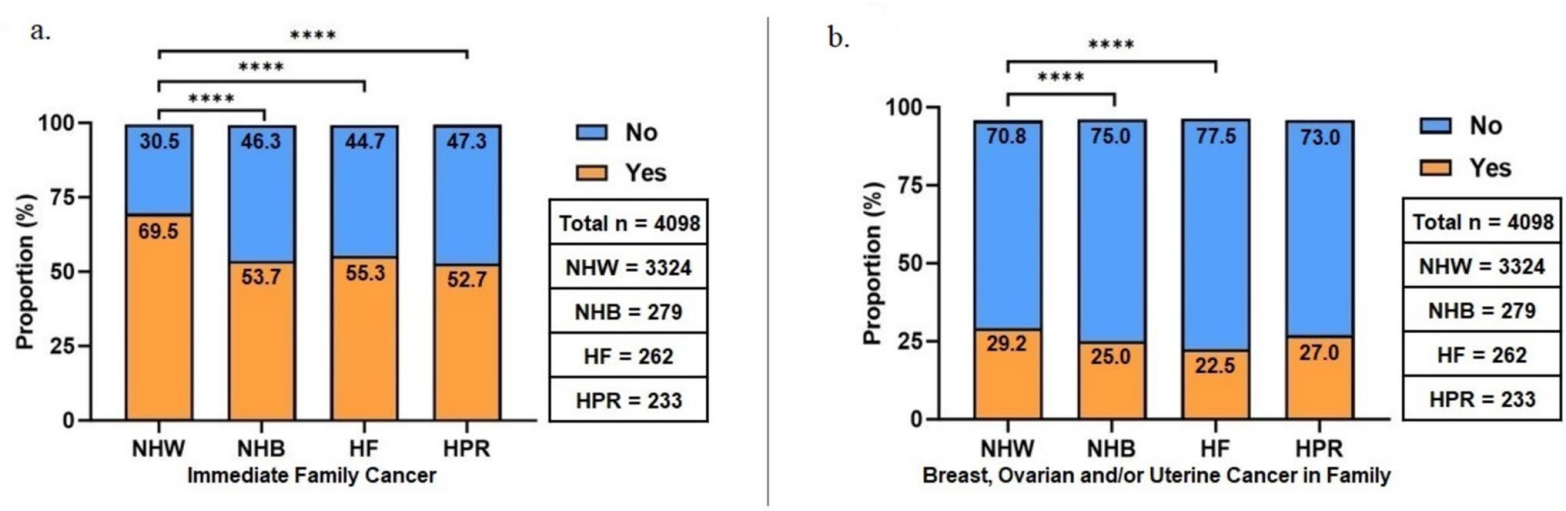
Patient family history of cancer by cohort: any cancer type (**a**), breast, ovarian, and/or uterine cancer (**b**). Significant group differences were determined via chi-squared test and are indicated as ****p < 0.0001

Data in Fig. 3a reveal that 69.5% of NHW women report that one or more of their first-degree relatives (parents, siblings, children) have been diagnosed with cancer. This is significantly higher than each of the other cohorts, with 53.7% of NHB patients (*p* < 0.0001), 55.3% of HF patients (*p* < 0.0001), and 52.7% of HPR patients (*p* < 0.0001) reporting cancer among their immediate family. As demonstrated in Fig. 3b, this trend persists when considering familial breast, ovarian, and uterine cancer only, with NHW women reporting the highest rate of these cancers in their first-degree relatives at 29.2%, which is significant relative to NHB (25.0%, *p* < 0.0001) and HF (22.5%, *p* < 0.0001) groups. A full description of family cancer history data can be found in Supplemental Table 3.

### Age at Diagnosis and Body Mass Index (BMI)

**Fig. 4:**
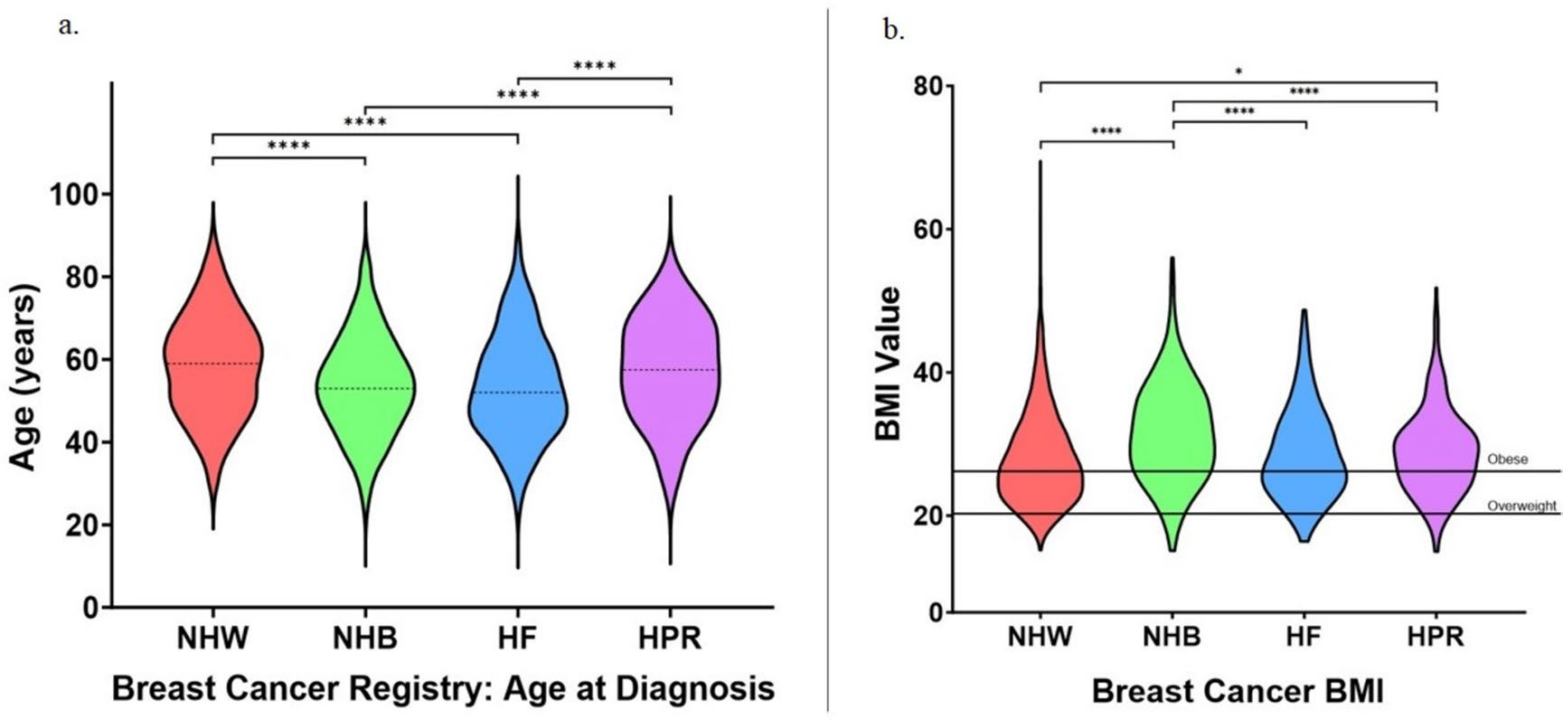
Violin plots of breast cancer patient age at diagnosis by cohort (**a**) and of breast cancer patient BMI at diagnosis by cohort (**b**). Significant group differences were determined via the Tukey test and are indicated as *p < 0.05, ****p < 0.0001

Figure 4 presents violin plots describing age at breast cancer diagnosis and BMI trends in our patient populations. Figure 4a focuses on age at breast cancer diagnosis by cohort. Cancer Registry data demonstrates that the NHB and HF cohorts are younger at breast cancer diagnosis (median ages of 53 and 52, respectively) compared to NHW and HPR women (median ages of 59 and 58, respectively). On pairwise comparison, each of the younger cohorts was significantly distinct from each of the older cohorts (*p* < 0.0001, on all four comparisons). A full description of the age of breast cancer diagnosis data is in Supplemental Table 5. Figure 4b presents patient BMI at breast cancer diagnosis by cohort. The mean and median BMI of the NHW, HF, and HPR groups fall within the overweight category (25.0–29.9), while the mean and median BMI for the NHB group fall into the obese category of 30.0 or greater. Figure 4b reveals the NHB cohort has a significantly higher BMI than each of the other cohorts on pairwise comparison (*p* < 0.0001). Among the other three groups, HPR women reported significantly higher BMIs than NHW women (*p* < 0.05). A full description of BMI data can be found in Supplemental Table 4.

### Tumor Characteristics

Analysis of hormone receptor data indicates that among our cohorts, NHB women most commonly (27.2%) and NHW women least commonly (11.6%) presented with the aggressive HR − disease. These relationships are significant on pairwise comparisons between NHW-NHB (*p* < 0.0001), NHB-HF (*p* < 0.0001), and NHB-HPR (*p* < 0.05). No significant differences were found in the proportions of triple negative tumors between the HPR and HF groups. Conversely, HR + /HER2 − subtypes are least common in NHB women (56.6%) and most common in NHW women (73.4%). These relationships are significant on pairwise comparisons between NHW-NHB (*p* < 0.0001), NHW-HF (*p* < 0.05), and NHW-HPR (*p* < 0.0001). The HPR population most commonly had HR + /HER2 + tumors (14.5%), with significance compared to the HF (11.2%, *p* < 0.0001) and NHW (10.4%, *p* < 0.05) populations. Very few HPR women (3.5%) presented with HR − /HER2 + breast cancer, which was significantly lower than NHB (5.8%, *p* < 0.05) women. A more complete description of hormone receptor data can be found in Supplemental Table 6.

**Fig. 5:**
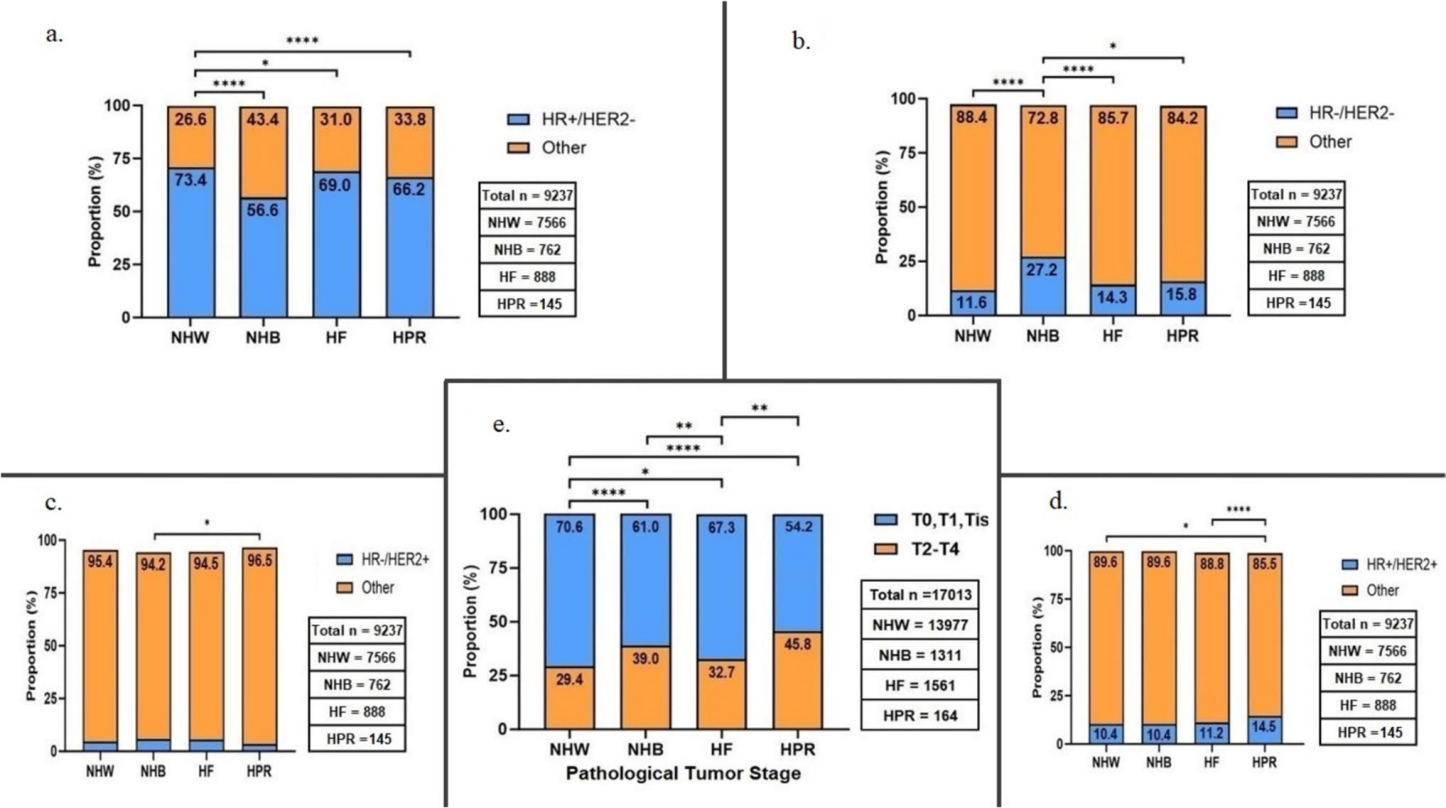
Hormone receptor data (**a**, **b**, **c**, **d**) and pathological tumor stage (**e**) by cohort. Significant group differences were determined via chi-squared test and are indicated as: *p < 0.05, **p < 0.01, ****p < 0.0001

In addition to HR and HER2 analyses, pathological tumor stage comparisons were made among the cohorts. Cohorts were subdivided based on pathologic tumor stage from Cancer Registry data. The first group includes pathological stages T1, T0, and Tis (in situ). The second group included patients presenting with pathological stages T2, T3, and T4 (and all substages within each group). NHW women (29.4%) are significantly less likely to have T2 + tumors at diagnosis than NHB (*p* < 0.0001), HF (*p* < 0.05), and HPR (*p* < 0.0001). Half (45.8%) of the HPR group have T2 + tumors as compared to only 29.4–39.0% of the other groups. Pairwise group comparison demonstrates the HPR are significantly more likely than NHW (*p* < 0.0001) and HF (*p* < 0.01) to present with T2 + tumors. As depicted in Supplemental Table 7, 8.3% of NHB women present with metastatic disease, which is significantly higher than NHW women (5.8%, *p* < 0.05) and HF women (5.4%, *p* < 0.001), but shows no significant difference relative to HPR women (3.4%). Similarly, Supplemental Table 7 shows that NHB women had the highest frequency of node-positive disease (41.7%) which was significantly higher than NHW (*p* < 0.0001, 30.7%) and HF (*p* < 0.0001, 33.5%) women and insignificant when compared to those in the HPR cohort (33.7%) (Fig. 5).

### Five-Year Survival

The NHW and HF groups had a significantly greater chance of 5-year overall survival than their NHB and HPR counterparts (Table 1, Supplemental Fig. 3 and Supplementary Table 8). When comparing each of our study cohorts to their corresponding race and ethnicity cohort in the Surveillance, Epidemiology, and End Results (SEER) program database, it was found that all three Moffitt-derived cohorts performed better than the associated SEER cohort with varying significance (not shown). The HPR population, however, had a worse 5-year survival rate than the corresponding SEER cohort, though this result was not significant. Kaplan–Meier analysis of survival outcomes between the cohorts can be found in Supplemental Fig. 3. Supplementary Table 8 reveals that after adjustments by age, stages, and HR status HPR (2.81, *p* < 0.001) and NHB (HR 1.51, *p* = 0.0155) still have statistically significantly worse survival compared with NHW patients.

## Discussion

In this analysis of comprehensive data from breast cancer patients from diverse backgrounds, we uncovered significant and impactful clinical outcome differences supporting the need for increased efforts to reduce cancer-related health disparities. This study compares data from NHW, NHB, and H/L patients from Florida (HF) and Puerto Rico (HPR). These analyses provide unique insight into the disparities experienced by cancer patients in the Ponce Health Region of southern Puerto Rico, resulting in older age and more advanced tumors at diagnosis, as well as worse survival compared not only to NHW patients but also to their H/L counterparts who live in FL. Based on known, previously described disparities in cancer outcomes we expected the NHW and NHB groups to serve as a best-case and worst-case reference group, respectively. Our study provides strong evidence indicating that Puerto Rican breast cancer patients experience similar and even worse clinical outcomes as NHB in the US. According to the 2020 US Census Bureau (www.census.gov), Puerto Ricans (at almost 20%) represent the second largest Hispanic subgroup (behind Mexicans who represent 50%) in Florida. Yet, there have been few efforts to compare clinical outcomes related to cancer that could tease out potential differences among Hispanic sub-groups.

Women with breast cancer living in Puerto Rico had a sociodemographic profile characterized by lower levels of college education and formal employment, with Puerto Rican women being three and seven times as likely to be homemakers as Hispanic women in Florida and NHW women, respectively. This demographic profile may synergistically affect healthcare seeking behaviors and amplify barriers to accessing medical care, leading to worse clinical outcomes.

The OB-GYN profiles reveal that both groups of H/L women had a younger age at menarche, in accord with other studies [35, 36]. Early menarche is a known risk factor for breast cancer since it leads to longer lifetime exposure to estrogen [37]. We also observed that the HPR group more frequently had three or more live births than the NHW and HF groups. Higher parity has been associated with a reduced incidence of breast cancer, with the greatest effect on the risk of developing a luminal subtype [38, 39]. The proportion of mothers who ever breastfed was significantly lower among HPR women compared to the other groups. Breastfeeding is generally associated with a reduced risk of breast cancer overall [39, 40], including a reduced risk of triple-negative disease [41]. In summary, we observe a mix of positive and negative risk OB-GYN factors in the Puerto Rican women.

Family cancer history, especially history of breast cancer, is a well-characterized risk factor in breast cancer [42]. Our data show that women in the NHW cohort report the highest rates of family history of cancer and familial female breast, ovarian, and/or uterine cancers, though the latter is only significant relative to NHB and HF groups. Although these observations seem counterintuitive given the better outcome data for NHW patients, it is noted that generally, the breast cancer incidence is higher in this group, whereas mortality is lowest [42]. We also hypothesize that these self-reported data may reflect a greater cancer awareness and earlier healthcare seeking behaviors in the NHW group.

The NHB and HF cohorts of our study are ~ 5–7 years younger at breast cancer diagnosis compared to NHW and HPR women. The observation that HF are diagnosed at a younger age than HPR was surprising and suggested that women in this group might be underdiagnosed or diagnosed at a later stage. Interpreting these observations in the light of pathological stage at diagnosis findings suggest that HPR are older at diagnosis, corresponding to a more advanced tumor stage. This finding might reflect reduced cancer awareness, less attention to screening, or barriers to receiving high-quality care. Unfortunately, the NHB group is diagnosed at a younger age and later stage suggesting that awareness and surveillance may be high, but the disease is more aggressive. Obesity is one of the most important modifiable risk factors related to cancer incidence, mortality, and survival [42, 43]. Our data reflects the well-established observation that NHB breast cancer patients present with higher BMI than NHW patients [44], while also showing that it is significantly higher compared to HF and HPR patients. Our data also reveal that HPR women have a slight, but statistically significant, elevation in BMI relative to NHW women.

Triple-negative breast cancer has the worst prognosis of the HR/HER2 subtypes, and its prevalence among NHB patients is thought to contribute to their greater breast cancer mortality. Our study demonstrates that the NHB group is much more likely to have triple-negative disease at breast cancer diagnosis (27% vs. 12–16% in the other groups). We conclude that the frequency of triple-negative breast cancer in our two H/L cohorts is not a major factor in survival disparities, in agreement with previous studies [45]. The NHB patients in our study also experienced the greatest frequency of node positivity and disease metastasis, with significance relative to their NHW and HF counterparts but no significance relative to HPR patients, which continues to emphasize the severity of disease in these patient populations. Analysis of overall survival for each of the four cohorts produced the most striking findings of our study: it showed that HPR and NHB breast cancer patients have reduced overall survival relative to NHW and HF patients. This finding is in accord with the observations that both NHB and HPR present more often with pathological stage 2 tumors or higher. These results likely represent the combined effects of multiple factors, some that were measured in this study and others that would require additional in-depth investigations.

The HPR breast cancer cohort represents an under-served, socioeconomically disadvantaged, and under-researched population [46], evidenced by the higher cancer mortality rates in the island. Disaggregating the data from H/L patient subgroups based on residence, uncovered impactful findings and insights into the inequities faced by cancer patients on the island. This population exhibits characteristics such as older age and more advanced tumors at diagnosis, coupled with inferior survival rates not only in comparison to NHW patients but also relative to their Hispanic counterparts residing in Florida. These findings challenge common assumptions by providing evidence that breast cancer patients in Puerto Rico endure comparable, if not more adverse, clinical outcomes than those experienced by NHB patients in the US.

What may be responsible for the poor survival outcomes in HPR with breast cancer? Besides the major role that socio-economic factors play in increasing poor survival rates in NHB and Hispanics [12], some biological drivers of poor outcomes are inherited in West African descendants (Caribbean-H/L, Black-HF, and NHB) from women native to West Africa. NHB have in general 90% West African genome, Puerto Ricans 21%, Hispanics from Chicago 18%, while H/L from other regions are 7% or less [47]. West African descendants tend to have breast tumors that are detected at younger ages, higher stages and grades, less likelihood of being localized, and aggressive breast cancer subtypes such as basal/ER-PR- subtypes [22, 29, 47–50]. Differences between NHW and NHB women with breast cancer are reflected at the molecular level, including higher expression of mitotic kinases, alterations in the p53 and the BRCA1 networks, less PI3C mutations, higher levels of aneuploidy and prevalent immune and basal signatures in NHB women with breast cancer [49, 51–54]. In future work, we intend to use clinical, molecular, epidemiological, and healthcare utilization data to explore at a deeper level the individual, community, and systemic underpinnings of this significant and pernicious cancer health disparity in HPR.

## Supplementary Information

Below is the link to the electronic supplementary material.

Supplementary file1 (DOCX 970 KB)

## Data Availability

Data supporting the findings of this study are available upon request.

## Abbreviations

ANOVA: Analysis of variance
BMI: Body mass index
ER: Estrogen receptor
HER2: Human epidermal growth factor receptor 2
HF: Hispanics in Florida
H/L: Hispanic/Latino
HPR: Hispanics in Puerto Rico
HR: Hormone receptors
IRB: Institutional Review Board
MCC: Moffitt Cancer Center
NCI: National Cancer Institute
NHB: Non-Hispanic Black
NHW: Non-Hispanic White
PACHE: Partnership to Address Cancer Health Equity
PHSU: Ponce Health Sciences University
PR: Progesterone receptor
PRBB: Puerto Rico Biobank
PRCCR: Puerto Rico Central Cancer Registry
SEER: Surveillance, Epidemiology, and End Results
DCCPS: Division of Cancer Control and Population Sciences
USA: United States
