# Supplementary material for: Worse Clinical and Survival Outcomes in Breast Cancer Patients Living in Puerto Rico Compared to Hispanics, Non-Hispanic Blacks, and Non-Hispanic Whites from Florida
Source: J Racial Ethn Health Disparities. 2024 Nov 14;13(1):130–40. doi: 10.1007/s40615-024-02232-5 (PMC12281488; doi:10.1007/s40615-024-02232-5)
Supplement: Supplementary file 1 — Supplementary file1 (DOCX 970 KB) [file 40615_2024_2232_MOESM1_ESM.docx]

**Supplemental Figures and Tables**

**Worse clinical and survival outcomes in breast cancer patients living in Puerto Rico when compared to Hispanics, non-Hispanic Blacks, and non-Hispanic Whites from Florida**

Journal of Racial and Ethnic Health Disparities

Abigail E. Lantz, Ryan Gebert, Jiannong Li, Jose A. Oliveras, Edna R. Gordián, Jaileene Perez-Morales, Steven Eschrich, Dung-Tsa Chen, Marilin Rosa, Julie Dutil, Harold I. Saavedra, Teresita Muñoz-Antonia, Idhaliz Flores, and W. Douglas Cress

**Corresponding Author**

W. Douglas Cress, PhD

Affiliations: Puerto Rico Biobank, H. Lee Moffitt Cancer Center & Research Institute, Tampa, Florida and the Ponce Health Sciences University, Ponce, PR; Department of Molecular Oncology, H. Lee Moffitt Cancer Center & Research Institute, Tampa, FL

Email: douglas.cress@moffitt.org

Table of Contents

[Supplemental Figures 2](#_Toc165022816)

[Supplemental Tables 5](#_Toc165022817)

# Supplemental Figures


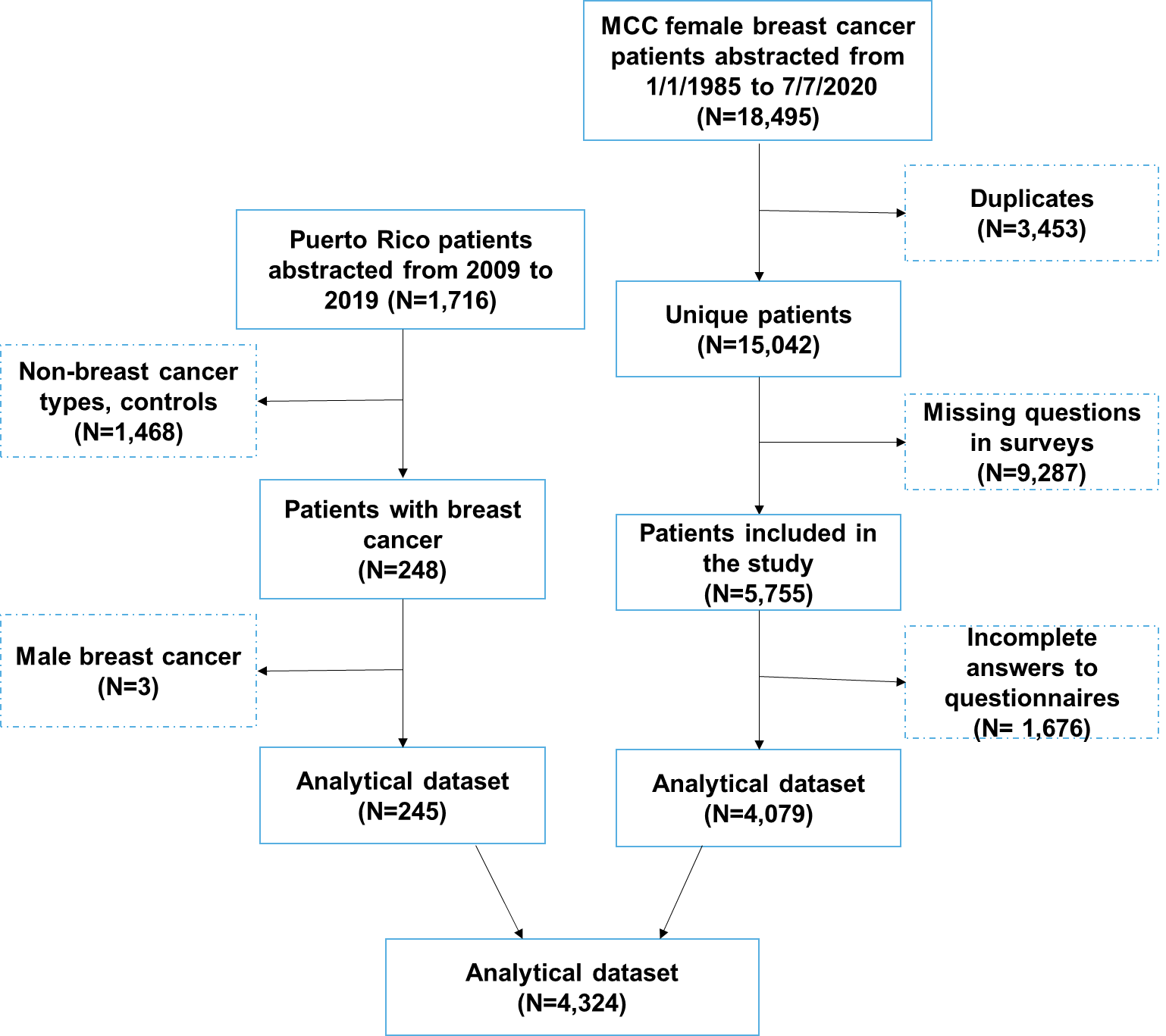


**Supplemental Fig. 1** Inclusion/Exclusion Flowchart of Patient Questionnaire Data


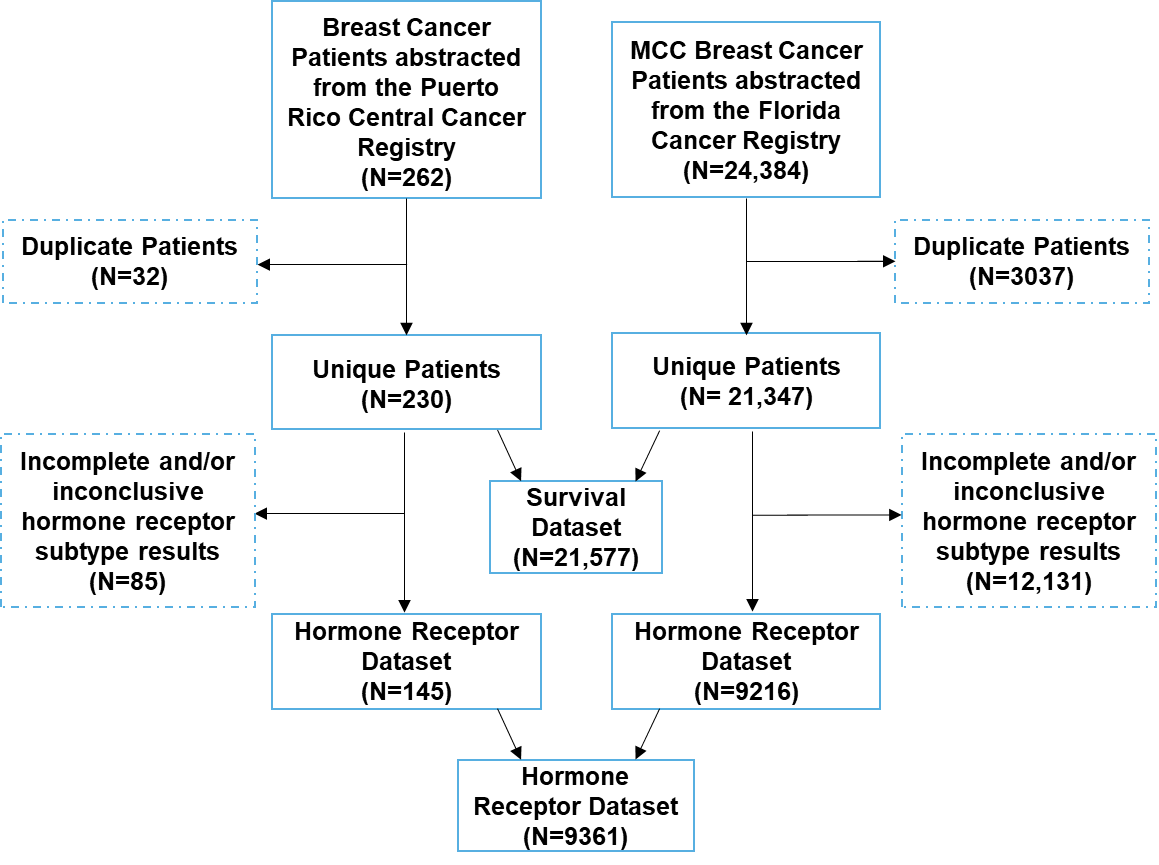


**Supplemental Fig. 2** Inclusion/Exclusion Flowchart of Cancer Registry Data


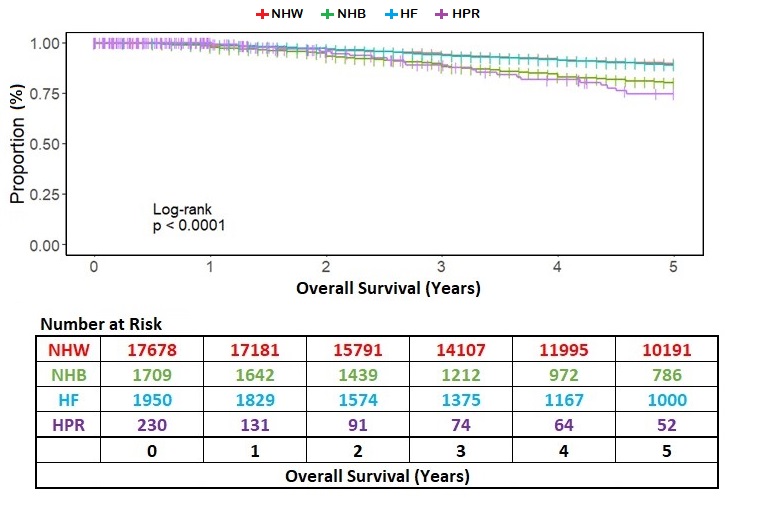


**Supplemental Fig. 3** Kaplan-Meier Curve: Survival by Cohort

# Supplemental Tables


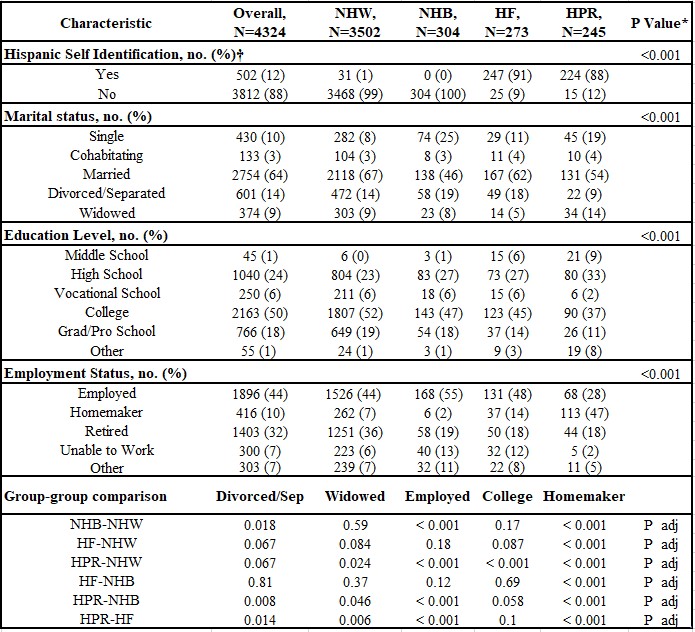
Determined using the Chi-squared test, †Blank responses were excluded, Benjamini & Hochberg Adjustment

**Supplemental Table 1** Patient Characteristics - Demographics

*Determined using Chi-squared test, †Blank responses were excluded, Benjamini & Hochberg Adjustment


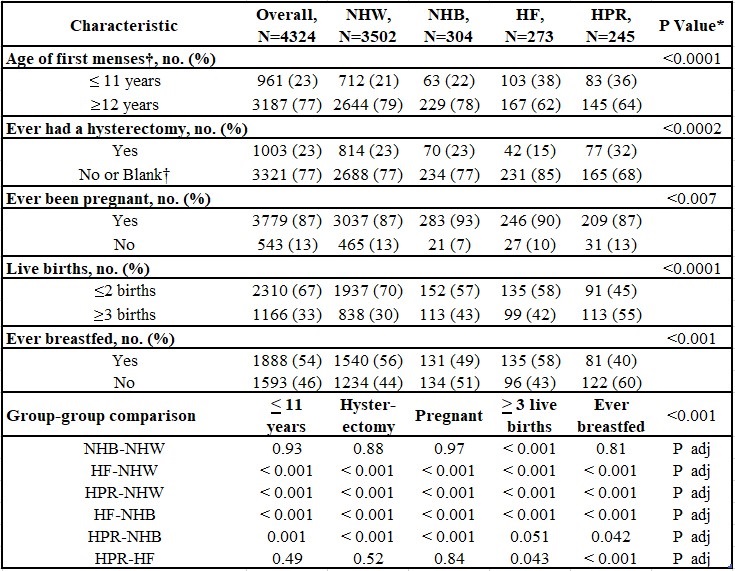


**Supplemental Table 2** Patient Characteristics - Ob-Gyn Variables


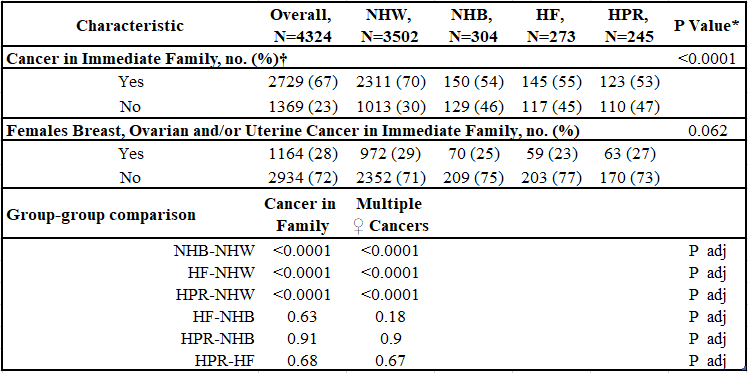
*Determined using Chi-squared test, †Blank responses were excluded, Benjamini & Hochberg Adjustment

**Supplemental Table 3** Family Cancer History Characteristics


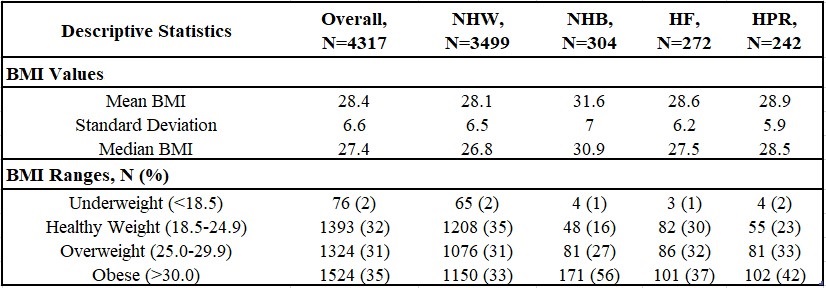
†Blank responses were excluded, Benjamini & Hochberg Adjustment

**Supplemental Table 4** BMI Descriptive Statistics by Cohort.


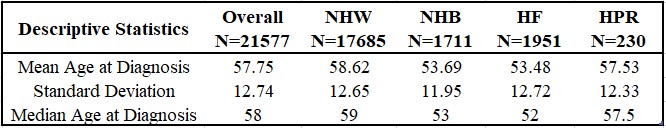
†Blank responses were excluded, Benjamini & Hochberg Adjustment

**Supplemental Table 5** Age at Diagnosis Descriptive Statistics by Cohort.


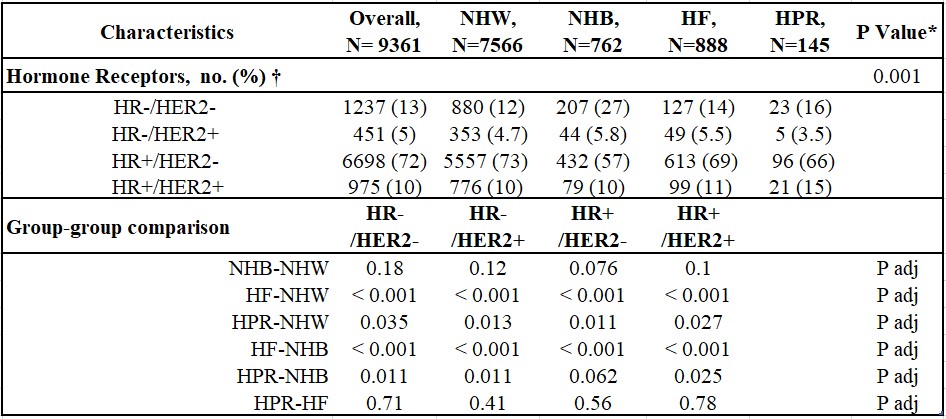
Determined using the Chi-squared test, †Blank responses were excluded, Benjamini & Hochberg Adjustment

**Supplemental Table 6** Hormone Receptor Descriptive Statistics by Cohort.


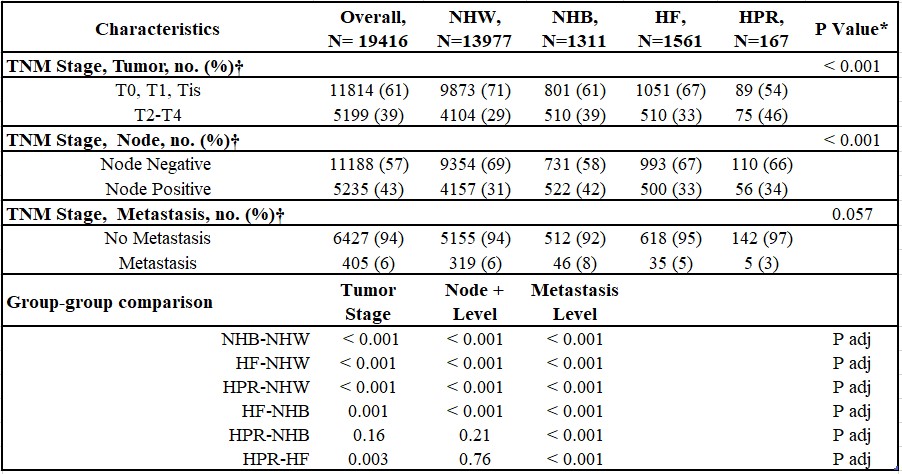
Determined using the Chi-squared test, †Blank responses were excluded, Benjamini & Hochberg Adjustment

**Supplemental Table 7** Pathological Stage Descriptive Statistics by Cohort.

| **Variable** | **Level** | **Hazard Ratio**  **(95% confidence interval)** | **P-value (Wald)** |
| --- | --- | --- | --- |
| **Cohort** | | | |
|  | NHW | 1 (Reference) | - |
|  | NHB | 1.51 (1.08, 2.10) | **0.0155** |
|  | HF | 0.93 (0.58, 1.48) | 0.7520 |
|  | HPR | 2.81 (1.84, 4.28) | **<0.001** |
| **Hormone Receptor Status** | | | |
|  | Negative | 1 (Reference) | - |
|  | Positive | 0.42 (0.33, 0.54) | **<0.001** |
| **T-Stage** | | | |
| T0, T1, Tis | Low Risk | 1 (Reference) | - |
| T2-T4 | High Risk | 2.16 (1.68, 2.79) | **<0.001** |
| **N-Stage** | | | |
|  | Node Negative | 1 (Reference) | - |
|  | Node Positive | 2.10 (1.62, 2.71) | **<0.001** |
| **M-Stage** | | | |
|  | Non-metastatic | 1 (Reference) | - |
|  | Metastasis | 3.33 (2.37, 4.69) | **<0.001** |
| **Age at Diagnosis** |  | 1.02 (1.01, 1.03) | **<0.001** |

**Supplemental Table 8** Cox proportional hazards model.
